# Maternal history of childhood sexual abuse and preterm birth: an epidemiologic review

**DOI:** 10.1186/s12884-015-0606-0

**Published:** 2015-08-15

**Authors:** Adaeze C. Wosu, Bizu Gelaye, Michelle A. Williams

**Affiliations:** Department of Epidemiology, Harvard T.H. Chan School of Public Health, 677 Huntington Ave, K505F, Boston, MA 02115 USA

## Abstract

**Background:**

History of childhood sexual abuse (CSA) is highly prevalent with as many as one in four American women being victims. Exposure to CSA or other early life traumatic experiences has been associated with adverse reproductive and pregnancy outcomes. However, the effects of CSA on preterm delivery (PTB), a leading cause of neonatal mortality, remain poorly understood. The objectives of this review are (i) to synthesize the available research investigating the relationship between maternal history of childhood sexual abuse (CSA) and preterm delivery (PTB); (ii) to provide suggestions for improving future research on this topic; and (iii) to highlight implications for clinical practice and public health.

**Methods:**

Relevant articles were identified through searches of four electronic databases (PubMed, CINAHL, Web of Science Core Collection and BIOSIS Online) for studies published before March 2014, as well as through reviewing references of published articles.

**Results:**

A total of six studies published from 1992 to 2010 were included in this review. Overall, findings were inconsistent. Three studies reported statistically significant associations of CSA with PTB (<37 weeks gestation) or shorter mean gestational age at birth. Women with a history of CSA had 2.6 to 4.8-fold increased odds of PTB as compared with women without a history of CSA. Three other studies did not observe statistically significant differences in rates of PTB or mean gestational age at birth in relation to a history of CSA.

**Conclusions:**

Available evidence on this topic is sparse and inconsistent, and limited by a number of methodological challenges. Given the ubiquity of CSA, as well as the clinical and public health significance of PTB, more rigorously designed epidemiologic studies on the association between CSA and PTB are warranted.

**Electronic supplementary material:**

The online version of this article (doi:10.1186/s12884-015-0606-0) contains supplementary material, which is available to authorized users.

## Introduction

### Definitions, prevalence and correlates of CSA

Childhood sexual abuse (CSA) is a major public health problem with serious immediate and long-term health consequences [1–3]. Although significant variation exists in its definition, CSA is generally recognized as the involvement of a child in sexual activity that is not developmentally appropriate, that he or she does not fully comprehend or is unable to give consent to, by an individual who by age or development is in a relationship of responsibility, trust or power to the child. CSA may involve use of manipulation, coercion, threats or violence to engage a child in sexual activity [4].

Generally, CSA is characterized into three broad categories: (a) *non-contact sexual abuse* (e.g. exhibitionism, indecent exposure, sexual harassment or voyeurism); (b) *contact sexual abuse without penetration* (e.g. non-genital fondling, kissing, or genital touching); and (c) *contact sexual abuse with penetration* (e.g. anal, oral, or vaginal intercourse) is recognized as the most severe. In addition, CSA may be characterized in terms of frequency, duration, age of onset of abuse, and relationship of the victim to the perpetrator [5].

Recent prevalence estimates of CSA, summarized in meta-analyses and multi-country studies confirm high global prevalence, with markedly higher prevalence among girls as compared with boys; see Table 1 for a summary of CSA estimates from recent studies [5–10]. Briefly, in their study of student and community samples from 22 countries, Pereda *et al.* reported that the average prevalence of CSA was 19.7 % for girls and 7.9 % for boys [10]. These figures were corroborated by other investigators who reported global CSA prevalence estimates of 7.6 % for boys and 18 % for girls [7]. It is important to note that CSA rarely occurs as a solitary episode but appears to consist of continued sexual victimization and maltreatment. CSA typically co-occurs with one or more types of childhood maltreatment (i.e., child neglect, physical abuse, and emotional abuse) [5, 11].

**Table 1:** Summary of global prevalence of CSA

First author (year)	Definition of CSA	Studies included	Prevalence estimates		
			Overall	Boys	Girls
Andrews (2004) [5]	Contact and non-contact (age varied across studies; upper limit was 18 years)	513 articles or reports	Range 3.8–67.7 %	Range 3.8–35 %	Range 8.4–67.7 %
WHO multi-country study (2005) [9]	Unwanted or forced sexual activity before age 15 years	24,058 individuals in 15 sites in 10 countries around the world	–––	–––	Range 1–21 %
Pereda (2009a) [6]	Contact and non-contact (age varied across studies; upper limit was 18 years)	38 independent articles representing 21 countries	Range 0–60 %	Range 0–60 %	Range 0–53 %
Pereda (2009b) [10]	Contact and non-contact (age varied across studies; upper limit was 17 years)	65 articles, covering 24 countries	N/A	7.9 % (95 % CI 6.0–10.3 %)	19.7 % (95 % CI 16.7–23.0 %)
Stoltenborgh (2011) [7]	Contact and non-contact (age varied across studies; upper limit was 18 years)	331 independent samples from 217 publications	11.8 % (95 % CI 10.0 – 13.8 %)	7.6 % (95 % CI 6.6–8.8 %)	18 % (95 % CI 16.4–19.7 %)
Barth (2013) [8]	Contact and non-contact (age varied across studies; upper limit was 19 years)	55 studies from 22 countries	Range 3–31 %	Range 3–17 %	Range 8–31 %

*Abbreviations*: *CSA* Childhood sexual abuse, *WHO* World Health Organization

The prevalence of CSA in hospital and clinic based studies of pregnant women, primarily from high-income countries, range from 3.2 to 32.2 % [12–22]. As with studies conducted among non-pregnant individuals, much of the heterogeneity in CSA prevalence estimates are attributable to differences in operational definitions of CSA, differences in study setting, study design, sampling methods and sample size. For example, Sørbø *et al.* observed CSA prevalence of 7 % using one question to screen for CSA: “have you been forced to have sexual intercourse (as a child, under 18 years old)?” [13], whilst Yampolsky *et al.* observed a prevalence of 32.2 % using the 14-item Childhood Sexual Assaults Scale [12].

### CSA and health outcomes

The relation of CSA with a range of health outcomes has been well documented among men and women. Investigators have reported strong associations of CSA with psychiatric disorders including post-traumatic stress disorder (PTSD) [2], depression [23], suicidal behavior [1, 2], and substance abuse [24, 25]. Investigators have also described dose–response relationships between CSA severity and worsening psychiatric health. For example, the number of suicide attempts was shown to increase with frequency of CSA (β = 0.56, SE = 0.23, *p* <0.05) and with use of force during CSA (β = 1.13, SE = 0.57, *p* < 0.05) among men [26]. Similarly, CSA involving intercourse was found to be more strongly associated with psychiatric and substance use disorders as compared with CSA that did not involve intercourse [24].

CSA is linked to increased odds of a host of adverse reproductive characteristics in women including early age at menarche [27, 28] and adolescent pregnancy [29]. Among pregnant women, history of CSA has been associated with psychiatric disorders [30, 31], physical, sexual, or emotional abuse during pregnancy [30], and lifestyle risk behaviors such as cigarette smoking [30, 32], all of which may endanger the health of both mother and her developing fetus.

### Definition, prevalence and correlates of preterm birth

Preterm birth (PTB), defined as birth prior to the completion of 37 weeks gestation [33], is the leading cause of neonatal mortality [34]. Globally, approximately 15 million infants (i.e., 10 % of all births) are delivered preterm and about 1 million of these infants die as a result of their prematurity [34]. Maternal risk factors associated with PTB include cigarette smoking [35], previous preterm birth [36], infection [37], preeclampsia [38], obesity [39], psychiatric disorders [40, 41], psychotropic medication use [42] and exposure to intimate partner violence [43].

Although the majority of preterm births occur in low and middle income countries [34], PTB remains a significant problem in developed countries. In the United States, 1 in 8 infants is born preterm, although PTB rates seem to be on the decline (from 12.8 % in 2006 to 11.6 % of all births in 2012) [33, 44]. Preterm birth is associated with enormous social and economic burdens as outlined in a report from the Institute of Medicine [45]. The report estimated the maternal birth costs, early intervention services, four disabling conditions occurring from preterm birth, and lost household and labor market productivity costs related to PTB to be at least $26.2 billion (or $51,600 per infant born preterm) in 2005 [45]. Compared with term infants, preterm infants are at an increased risk for immediate and long-term health problems [46, 47]. Notably, an accumulating literature now documents increased risk of later-life cardiovascular-related conditions for mothers of preterm infants [48–50].

Despite its high prevalence, the etiology of PTB is not well understood [45]. However, some investigators suggest that early life stress, such as CSA, may be associated with PTB [51]. Given the importance of CSA and PTB as two public health conditions with major consequences for maternal and child health, our objectives in conducting this review are: (i) to synthesize the limited available research investigating the relationship between maternal history of childhood sexual abuse (CSA) and preterm birth (PTB); (ii) to provide suggestions for improving future research on this topic; and (iii) to highlight implications for clinical practice and public health.

## Methods

### Search strategy

Relevant studies published before March 2014 were identified from four online databases (PubMed, Cumulative Index of Nursing and Allied Health Literature (CINAHL), Web of Science Core Collection and BIOSIS Online). A full list of search terms used can be found in Additional file 1: Table S1. We also reviewed references of retrieved publications to identify other potentially relevant articles reporting on the association between CSA and PTB.

### Selection criteria

The exposure of interest was maternal exposure to CSA, and the outcome of interest was preterm birth or gestational age at birth. To be included, studies had to: (1) define CSA as occurring sometime before age 18 years; (2) report quantitative associations between CSA and PTB or gestational age at birth; (3) be observational studies (cross-sectional, prospective cohort, retrospective cohort, and case–control studies); and (4) be full-length papers (conference abstracts, case studies, gray literature, editorials were excluded).

## Results

**Fig. 1:**
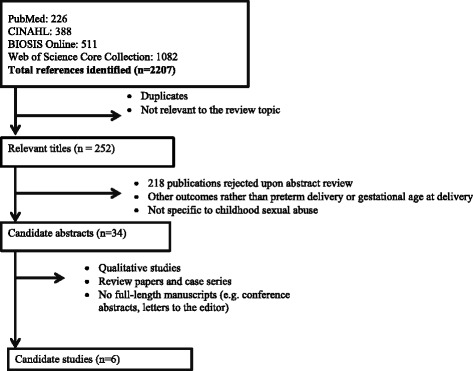
Flowchart showing selection of articles reporting on the relationship between CSA and preterm birth

The electronic database search identified 2,207 titles, of which 1955 were duplicates or rejected upon title review. The abstracts of the remaining unique 252 articles were read. Of these, 34 were selected for full-text screening (Fig. 1 summarizes the selection and screening process). Six full-length papers met the inclusion criteria and were included in this review (Table 2—papers in table are arranged by year of publication). The studies were published from 1992 to 2010 and in English. Four of the studies were from the United States while the other two were from Norway and Germany, respectively. Three studies used a prospective cohort design, two were retrospective cohort studies, and one was a case–control study. Findings were inconsistent, with only three studies reporting statistically significant positive associations of CSA with PTB or shorter mean gestational age at birth. In the sections below, we describe each study.

**Table 2:** Summary of original studies examining the association of maternal history of CSA with preterm birth or gestational age at birth

First author (year)	Country	Study design	Sample size	Recruitment	CSA definition	PTB definition	CSA and PTB findings
Jacobs (1992) [56]	United States	Retrospective	15 CSA-exposed, 13 controls	CSA-exposed women recruited from survivors’ group, mental health center and through therapists; controls recruited from evening psychology class taught in the community	CSA definition not specified	Mean gestational age at birth	No significant correlation between being a victim of CSA and gestational age at birth in the entire sample (r = 0.12, *p* > 0.05). However, the authors reported a positive correlation between gestational age at birth and maternal history of being sexually touched as a child (r = 0.34, *p* < 0.05).
Stevens-Simon (1994) [52]	United States	Prospective cohort	127 women	Participants in the Rochester Study of Adolescent Pregnancy	Physical or sexual abuse (<19 years)	<37 weeks gestation	CSA-exposed women had shorter mean gestational lengths (38.0 ± 3.4 weeks) compared with non-exposed (39.1 ± 1.7 weeks, *p* ≤ 0.05). PTB more common among exposed women (19.1 % vs. 4.7 %, *p* ≤ 0.05; OR = 4.76, 95 % CI: 1.34, 16.89)
Benedict (1999) [55]	United States	Prospective cohort	357 women	Prenatal clinics in a large university-based hospital; women interviewed at 28–32 weeks gestation	≥1 non-consensual and non-experimental contact or non-contact sexual episode (<18 years) by a perpetrator who was ≥5 years older than victim	<37 weeks gestation	No statistically significant association between CSA and gestational age at birth
Grimstad (1999) [32]	Norway	Case–control	82 women with low birth weight infants, 91 women with normal birth weight infants	Department of Obstetrics, the University Hospital of Trondheim	Adverse sexual experiences (<18 years)	Not defined	PTB prevalence similar for abused and non-abused women (36 % vs. 32 %, *p* = 0.68)
Noll (2007) [53]	United States	Prospective cohort	40 CSA-exposed, 31 controls	CSA-exposed girls were referred by CPS; unexposed girls were recruited through community advertisements	Substantiated contact sexual abuse perpetuated by a family member (between 6 and 16 years)	<37 weeks gestation	PTB risk increased among women with CSA vs. non-abused women (21 % vs. 11 %; OR = 2.80, *p* < 0.05)
Leeners (2010) [54]	Germany	Retrospective cohort	85 CSA-exposed, 170 controls	CSA-exposed women recruited through sexual abuse survivor centers in large cities; unexposed women were recruited through local kindergartens	Contact and non-contact sexual abuse (<18 years)	<37 weeks gestation	PTB risk increased among women with CSA history vs non-abused women (18.8 % vs. 8.2 %, *p* = 0.02; OR = 2.58, 95 % CI: 1.19 – 5.59)

*Abbreviations*: *CPS* Child protective services, *CSA* Childhood sexual abuse, *PTB* Preterm birth

Steven-Simons and colleagues analyzed data for 127 women who were <19 years old at the time of conception of pregnancy. History of current or prior physical or sexual abuse was obtained through interviews and was defined as answering “yes” to the question “Have you ever been physically or sexually abused?” or other related questions. Overall, 42 women (33 %) reported exposure to childhood physical or sexual abuse. The authors noted that abused women delivered approximately 1 week earlier, on average, as compared with non-abused women (mean gestational age at birth: 38.0 ± 3.4 vs. 39.1 ± 1.7 weeks, *p* ≤ 0.05). Also, the frequency of PTB was higher among abused women as compared with non-abused women (19.1 % vs. 4.7 %, *p* < 0.05; unadjusted OR = 4.76, 95 % CI: 1.34, 16.89). However, the authors reported that when controlling for maternal stress, depression, social support, and substance abuse, the association between abuse and gestational age at birth was not statistically significant (quantitative summary not reported) [52]. It is possible that some of these factors may be indirect effects of CSA. A major strength of this study is the use of various methods to substantiate infant gestational age at birth. However, inference from this study is limited by the lack of differentiation between childhood sexual and physical abuse, and lack of adjustment for age. Since the sample ranged from 12 to 18 years old, youngest members of the sample may have been at particularly high risk for preterm birth.

In a prospective cohort study of 40 women with CSA history and 31 without a history of CSA, Noll and colleagues reported that the odds of PTB was elevated among women with a history of CSA as compared with those who had no such history of abuse (21 % vs. 11 %; adjusted OR = 2.80, *p* < 0.05, adjusted for minority status and sibling number). Prenatal alcohol use, but not maternal cortisol concentrations, mediated this relationship [53]. Important strengths of this study included the prospective study design, and clear definition of CSA (i.e., contact sexual abuse by a family member that occurred while participants were between the ages of 6–16 years, and was reported to Child Protection Services), and major improvements in statistical analyses over preceding studies.

Recently, Leeners *et al.* conducted a retrospective cohort study of 85 women with CSA history who were attending sexual abuse support centers and 170 women without a history of CSA. CSA was defined as contact or non-contact sexual abuse prior to age 18 years and delivery information was ascertained from medical records. The investigators found that abused women were more likely to deliver preterm as compared with non-abused women (18.8 % vs. 8.2 %, *p* = 0.02; unadjusted OR = 2.58, 95 % CI: 1.19, 5.59) [54].

In contrast to the studies described above, three other studies did not observe significant associations of CSA with PTB or mean gestational age at birth. In a case–control study conducted in Norway, Grimstad and colleagues analyzed data for 82 women who delivered low birth weight (<2500 g) infants, and 91 women with normal birth weight (≥2500 g) infants. A positive history of CSA was determined on the basis of participants’ response to a single question as to whether they had negative sexual experiences prior to age 18 years. The authors did not observe a significant difference in the prevalence of preterm birth (definition not specified) among women with CSA history (36 %) and women with no CSA history (32 %, *p* = 0.68) [32].

Benedict and colleagues conducted a prospective cohort study to examine the association of CSA with depressive symptomatology, negative life events, and selected pregnancy outcomes, including gestational age at birth among 357 women aged ≥18 years. CSA was defined as ≥ 1 episode of non-consensual and non-experimental contact or non-contact sexual abuse prior to 18 years by a perpetrator ≥ 5 years older than the victim. However, if force was used, women were considered as CSA-exposed regardless of age difference with perpetrator. Thirty-seven percent of participants reported exposure to CSA and prevalence of PTB in the entire sample was 13 %. The authors reported no significant association of CSA with mean number of gestational weeks at birth or PTB observed (no quantitative summary was shown) [55]. Finally, in a small (*N* = 28) retrospective cohort study, Jacobs reported no statistically significant association between being a victim of CSA and PTB or gestational age at birth. However, the authors reported a positive correlation between gestational age at birth and maternal history of being sexually touched as a child (r = 0.34, *p* < 0.05). Due to a number of methodological challenges, one being an extremely small sample size, findings from this study, particularly those on the CSA sub-category of being sexually touched as a child, should be interpreted with caution [56].

### Limitations of available evidence

These studies on the association of CSA with PTB or gestational age at birth had some common limitations. First, the majority of studies were based on convenience samples with relatively small sizes (range from 28 to 357 women), which limited statistical power and hindered inferences that could be made from the studies. Second, there was significant heterogeneity across studies with regard to the definition of CSA, i.e., level of contact considered, mode of ascertainment of CSA history, and the maximum cutoff age for CSA (range from 16 to 19 years of age). These differences in the operational definition of CSA may have contributed to variations in CSA prevalence and observed associations of CSA with PTB and gestational age at birth. Third, the mode of ascertainment of PTB or gestational age at birth varied, from maternal self-report [56], to extraction from medical records [53–55], to ultrasound dating [32, 52]. Fourth, most of the studies were not specifically designed to determine the extent to which, if at all, maternal history of exposure to CSA is associated with PTB risk, rather they were secondary analyses of data collected for other purposes. Thus, most analyses of the CSA-PTB relationship were preliminary and did not adequately account for important confounding factors, mediators and modifiers such as maternal preconception and antepartum exposures to cigarette smoking, mood and anxiety symptomatology, and stressors such as intimate partner violence and sexual abuse in adulthood. Fifth, CSA histories were assessed retrospectively for the majority of studies, and so may have been subject to reporting errors. Lastly, all studies were from high-income countries (US, Norway and Germany). Thus, results may not be generalizable to women in low and middle-income countries.

### Hypothesized biological mechanisms

Pathophysiological mechanisms that may account for observed associations of CSA exposure histories with PTB are not well known. However, investigators have suggested that CSA, an early life stressor, contributes to psychological stress and promotes dysregulation of the hypothalamic-pituitary-adrenal (HPA) axis, one of two major neuroendocrine pathways activated in stress response [51]. HPA axis activation begins with discharge of corticotrophin-releasing hormone (CRH) from the hypothalamus, which then triggers the secretion of adrenocorticotropic hormone (ACTH) by the pituitary and the subsequent release of cortisol by the adrenal cortex [57]. CRH is also found in other sites including the placenta, ovaries, and adrenal glands [57, 58]. During pregnancy, maternal CRH concentrations rise due to increased CRH synthesis in the fetus, placenta and uterine lining, resulting in increases in maternal ACTH and cortisol concentrations [58].

In their review paper, Horan and colleagues propose that the trauma, stress and fear associated with CSA may stimulate enhanced CRH gene expression and chronic overproduction of CRH in the brain, making a woman susceptible to elevated placental CRH gene expression during pregnancy and consequently, increased risk of PTB [51]. This thesis is supported by animal studies that showed that infusion of CRH initiated early labor [59] and a Type I CRH receptor antagonist delayed parturition [60]. In humans, elevated third trimester placental CRH concentrations have been associated with increased risks of spontaneous PTB and/or fetal growth restriction [61–63]. These studies support CRH’s role in labor initiation, particularly its function as a placental clock that may regulate the length of human gestation [64]. CSA may also increase risk for PTB through mediators such as psychiatric disorders [40], obesity [25], and lifestyle factors such as alcohol use [53], and cigarette smoking [65], which have been shown to be associated with CSA [24, 30] and with increased risk for PTB [45]. As observed by Noll and colleagues (in one of the studies included in this review) maternal prenatal alcohol use was a mediator of the CSA-PTB relationship [53].

## Discussion

CSA is a highly prevalent early life stressor with wide-ranging immediate and long-term biological and psychological sequelae [5]. Among pregnant women, some investigators have documented associations of CSA history with cigarette smoking [30, 32], psychiatric disorders [31], and abuse during pregnancy [30], known risk factors for preterm birth. Preterm birth occurs in 10 % of all births globally, and has tremendous medical, economic, and health implications for mother, infant, and society at-large [45]. Available evidence suggest that maternal history of early life adversity may play a role in PTB [51]. However, only a limited number of studies have empirically examined associations of CSA, a common stressor in the lives of young girls and women, with PTB.

As reported in this review of the six published studies on the topic to date, only three studies observed statistically significant associations of CSA with PTB or shorter mean gestational age at birth [52–54]. Three other studies did not observe substantial or statistically significant associations [32, 55, 56], although one study reported higher prevalence of PTB for women with a history of CSA (36 %) compared to women with no such history (32 %) [32] and another study observed an increased gestational age at birth with one sub-category of women with a history of CSA [56]. Inferences from the majority of available studies on the CSA-PTB relationship are hindered by small sample sizes, and incomplete control of confounding factors. Of note, these studies did not adequately distinguish the effects of CSA from the effects of other forms of childhood maltreatment or trauma, or later-life abuse. CSA and PTB are common and have significant clinical and developmental consequences for mothers and children. There is need for longitudinal and rigorously designed studies to improve understanding of the CSA-PTB relationship. Here, we offer some considerations for improving future studies on this topic:

1. The use of detailed CSA questionnaires (e.g., The Childhood Trauma Questionnaire and The Sexual and Physical Abuse Questionnaire) in future CSA-PTB studies would allow for more uniform exposure collection efforts and capture of a wide range of CSA severities to enable nuanced analyses and interpretation of findings.
2. Studies should distinguish between sub-categories of preterm birth (i.e., extremely preterm, very preterm, and moderate to late preterm) to add greater specificity to the existing literature and further inform the development of clinical risk stratification and risk management protocols.
3. Longitudinal studies incorporating the use of biological samples may facilitate understanding of biological mechanisms and identify important mediators and modifiers of the CSA-PTB relationship.
4. Study design and analytical approaches to account for confounders and potential mediators (e.g., other forms of child maltreatment and trauma, adult traumatic experiences, socioeconomic status, prenatal cigarette smoking, illicit drug or alcohol use) will improve validity and precision of measured CSA-PTB associations.
5. Efforts should be made to quantify the contribution of CSA to PTB incidence in middle and low-income countries, particularly those countries with high prevalence of CSA. Such efforts would expand current knowledge and may be informative for PTB prevention in these settings.
6. Prevention of both CSA and PTB requires a multi-sectorial response. Prevention and risk management efforts should engage individuals from health services, educational, advocacy, institutions, with better recognition of the social, cultural and environmental milieu within which CSA and PTB occur.

## Conclusions

### Clinical and public health implications

In spite of the great strides that have been made towards elucidating the medical, environmental, psychosocial and genetic risk factors of PTB [45], this review underscores that much remains to be done to fully understand the contribution of CSA to PTB. Given (1) the high prevalence of history of CSA among women, (2) the relationships between CSA and adverse reproductive characteristics and risk behaviors during pregnancy, (3) the accumulating evidence linking PTB to adverse long-term maternal and child health outcomes, and (4) the observed associations of maternal history of CSA with PTB, identifying women with a history of CSA, and providing them with increased attention and care during pregnancy may be one important strategy for PTB prevention. Finally, women with a history of CSA may also benefit from additional education and intervention (e.g., psychosocial support) targeted at modifying health risk behaviors for better maternal and infant health outcomes.

## Additional file

Additional file 1: Table S1. Search terms used to identify relevant publications. (DOCX 14 kb)

## Abbreviations

ACTH: Adrenocorticotropic hormone
CI: Confidence interval
CINAHL: Cumulative index to nursing and allied health literature
CRH: Corticotrophin-releasing hormone
CSA: Childhood sexual abuse
HPA: Hypothalamic-pituitary-adrenal
OR: Odds ratio
PTB: Preterm birth
US: United States
WHO: World Health Organization
