# Supplementary material for: Maternal history of childhood sexual abuse and preterm birth: an epidemiologic review
Source: BMC Pregnancy Childbirth. 2015 Aug 15;15:174. doi: 10.1186/s12884-015-0606-0 (PMC4537581; doi:10.1186/s12884-015-0606-0)
Supplement: Additional file 1: Table S1. — Search terms used to identify relevant publications. (DOCX 14 kb) [file 12884_2015_606_MOESM1_ESM.docx]

**Supplementary Table 1. Search terms used to identify relevant publications**

| **PubMed: 226** |
| --- |
| ("child abuse, sexual"[mesh] OR "adult survivors of child abuse"[mesh] OR early life advers* [tiab] OR child trauma*[tiab]) AND (pregn*[tiab] OR preterm*[tiab] OR "premature birth"[mesh] OR "infant, premature"[mesh] OR prematur*[tiab] OR gestat*[tiab]) |
| **CINAHL: 388** |
| (MH (“Child abuse+” OR “Childhood Trauma+” OR “Early life adversity+” OR “Childhood sexual abuse+”)) OR (TI (child abuse OR child sexual abuse OR childhood trauma* OR early advers* OR history of sexual abuse OR adult survivors of child abuse)) OR (AB (child abuse OR child sexual abuse OR childhood trauma* OR early advers* OR adult survivors of child abuse))  AND  (MH (“Preterm birth+” OR “Preterm labor+” “Preterm delivery+” OR “Premature birth+” OR “Pregnancy Outcomes+” OR “Pregnancy Complications+” OR “Premature labor+” “Premature delivery+”)) OR (TI (prematur* OR preterm* OR pregnan* OR gestat* OR early labor)) OR (AB (prematur* OR preterm* OR pregnan* OR gestat* OR early labor*)) |
| **Web of Science Core Collection: 1082; BIOSIS: 511** |
| TS =((child sexual abuse OR childhood trauma questionnaire OR adult survivors of child abuse OR maternal history of sexual abuse OR early life adversity OR child trauma*) AND (pregn* OR preterm* OR gestat* OR early labor OR prematur*)) |
